# Magnetic Resonance Imaging and Its Effects on Metallic Brackets and Wires: Does It Alter the Temperature and Bonding Efficacy of Orthodontic Devices?

**DOI:** 10.3390/ma12233971

**Published:** 2019-11-30

**Authors:** Maria Francesca Sfondrini, Lorenzo Preda, Fabrizio Calliada, Lorenzo Carbone, Luca Lungarotti, Luisa Bernardinelli, Paola Gandini, Andrea Scribante

**Affiliations:** 1 Unit of Orthodontics and Paediatric Dentistry, Section of Dentistry—Department of Clinical, Surgical, Diagnostic and Paediatric Sciences, University of Pavia, 27100 Pavia, Italy; francesca.sfondrini@unipv.it (M.F.S.); lorenzo.carbone01@universitadipavia.it (L.C.); paola.gandini@unipv.it (P.G.); 2 Department of Clinical, Surgical, Diagnostic and Paediatric Sciences, University of Pavia, 27100 Pavia, Italy; CNAO Foundation Diagnostic Imaging Unit, National Center of Oncological Hadrontherapy (CNAO) Pavia, 27100 Pavia, Italy; lorenzo.preda@unipv.it; 3 Section of Radiology—Department of Clinical, Surgical, Diagnostic and Paediatric Sciences, University of Pavia, 27100 Pavia, Italy; fabrizio.calliada@unipv.it; 4 Department of Diagnostic and Interventional Radiology—Fondazione IRCCS Policlinico San Matteo, Pavia, 27100 Pavia, Italy; luca.lungarotti01@universitadipavia.it; 5 Section of Statistics—Department of Brain and Behavioral Sciences, University of Pavia, 27100 Pavia, Italy; luisa.bernardinelli@unipv.it

**Keywords:** dentistry, orthodontics, nuclear, magnetic, resonance, shear, bond, strength, temperature, adhesion, bracket, wire

## Abstract

Magnetic resonance imaging (MRI) is a widely used diagnostic technique. Patients wearing orthodontic appliances are often requested to remove their appliances, even when the MRI exam involves anatomical areas far from mouth, in order to avoid heating of the metal and detachment of the appliance. The purpose of the present investigation was to measure and compare temperature changes and orthodontic appliances’ adhesion to enamel after different MRIs. A total of 220 orthodontic brackets were bonded on bovine incisors and wires with different materials (stainless steel and nickel titanium). Moreover, various sizes (0.014″ and 0.019″ × 0.025″) were engaged. Appliances were submitted to MRI at two different powers (1.5 T and 3 T). The temperatures of brackets and wires were measured before and after MRI. Subsequently, the shear bond strength (SBS) and adhesive remnant index (ARI) scores were recorded. Statistical analysis was performed. After MRI, a significant increase in the temperature was found for both the brackets and wires in some groups, even if the mean temperature increase was clinically insignificant, as the temperature ranged between 0.05 °C and 2.4 °C for brackets and between 0.42 °C and 1.74 °C for wires. The MRI did not condition bracket adhesion in any group. No differences were reported when comparing the 1.5 T with 3 T groups. The ARI Scores were also significantly lower after MRI. The results of the present report show that, under MRI, orthodontic appliances present a low temperature rise and no debonding risk. Therefore, the removal of orthodontic appliance is not recommended routinely, but is suggested only in the case of a void risk or potential interference in image quality.

## 1. Introduction

Magnetic resonance imaging (MRI) is a non-invasive radiologic diagnostic technique that is widely used to assess lesions, particularly those involving soft tissues [1]. As this procedure does not involve the use of ionizing radiation, its applicability is wide and common, for both young and aged patients [2].

Under MRI, items are magnetized according to their magnetic susceptibility, and metallic devices produce a signal void that is visible in the radiographic image as a black spot [3]. Additionally, the magnetic field attracts metal objects that patients could accidentally wear during examination, resulting in patient injury and damage to the radiographic device [4]. For this reason, the first request of the radiologist to a patient before MRI is to remove any metal objects, even if they are far from the anatomical region to be examined [5].

The need to use MRI on patients wearing orthodontic appliances is not uncommon. During conventional orthodontic treatment, stainless steel brackets are usually bonded to the teeth, and metallic wires are engaged [6]. In these cases, the removal of orthodontic appliance is recommended to avoid image artifacts, unwanted bracket detachment, and an increase in the temperature of the brackets and wires [7,8]. However, to date, no clear guidelines are available for this process. In fact, the removal of orthodontic appliance, even for only a few days or hours, is time consuming, costly, and uncomfortable for both the patient and the clinician [9]. Moreover, this procedure could damage the enamel structure or lengthen treatment time [10].

Low metal or metal free orthodontic therapies offer a viable alternative [11,12]. Indeed, ceramic, fiber, composite, and other metal-free brackets are currently available on the market [13]. However, these materials are more breakable than metallic ones [14]. Moreover, these devices work with metallic wires and tubes, so the main problem remains partially unsolved. On the other hand, transparent removable devices (aligners) have shown excellent clinical results [15], but the lack of an active appliance permanently bonded to the teeth could lead, in some cases, to lower precision in certain movements [16]. For these reasons, stainless steel orthodontic appliances currently remain the golden standard in the majority of orthodontic treatments [17].

Therefore, the purpose of the present investigation was to evaluate the effects of MRI at two different powers (1.5 T and 3 T) on the temperature of the brackets and wires and on the bond strength and adhesive remnant index scores of orthodontic appliances. The first null hypothesis of this study is that there is no significant difference in the temperature among the various conditions tested. The second null hypothesis of the investigation is that there is no significant difference in the shear bond strength values among different groups. The third null hypothesis is that there is no change in the frequency distribution of the adhesive remnant index scores.

## 2. Materials and Methods

### 2.1. Specimen Preparation

The Unit Internal Committee Board approved the study. A total of 220 bovine lower incisors were collected and stored in thymol at 0.1% weight/volume [18]. The teeth were cleaned and randomly divided into 11 groups of 20 specimens each, as follows:

Group 1: No MRI—No Wire
Group 2: 1.5 T MRI—No wire
Group 3: 1.5 T MRI—0.014 inch stainless steel wire
Group 4: 1.5 T MRI—0.019 × 0.025 inch stainless steel wire
Group 5: 1.5 T MRI—0.014 inch nickel titanium wire
Group 6: 1.5 T MRI—0.019 × 0.025 inch nickel titanium wire
Group 7: 3 T MRI—No wire
Group 8: 3 T MRI—0.014 inch stainless steel wire
Group 9: 3 T MRI—0.019 × 0.025 inch stainless steel wire
Group 10: 3 T MRI—0.014 inch nickel titanium wire
Group 11: 3 T MRI—0.019 × 0.025 inch nickel titanium wire

**Figure 1:**
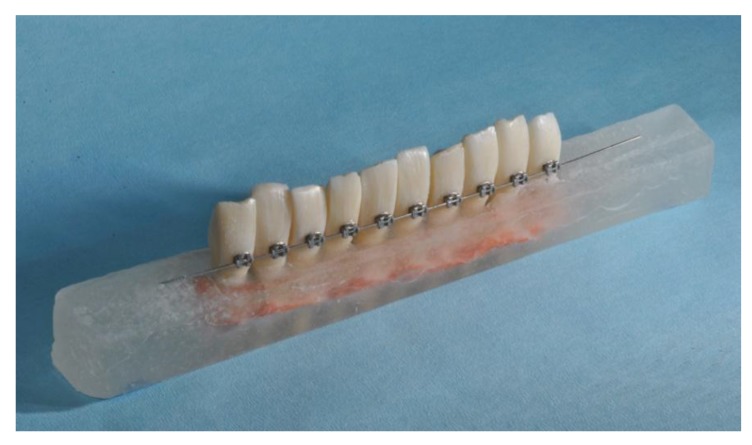
Block of 10 teeth ready for magnetic resonance imaging (MRI). The inter bracket distance was set at 5 mm. The appliance was bonded on the vestibular enamel, and the bracket base was parallel to the vestibular face of the resin block.

For each group, two blocks containing 10 teeth each (Figure 1) were prepared [19]. Incisors were reduced on their mesial and distal sides to allow an inter bracket distance of 5 mm. The vestibular enamel surface was kept parallel to the vestibular face of the resin blocks to facilitate correct bracket placement.

The materials (orthodontic wires, orthodontic brackets, and adhesive system) tested in the present investigations are listed in Table 1.

The bonding procedure involved conditioning of the enamel surface with orthophosphoric acid gel (Gerhò Etchant Gel, Gerhò Spa, Settequerce, Italy) for 30 s followed by washing and drying with an oil free air steam. Then, adhesive (Transbond XT primer, 3M Unitek Monrovia, CA, USA) was applied on the enamel and gently dried for 3 s. Resin (Transbond XT resin, 3M Unitek Monrovia, CA, USA) was applied on the bracket base, and then the bracket was squeezed onto the enamel. Excess resin was removed with a probe, and the adhesive was cured with a curing light (Starlight Pro, Mectron Medical Technology, Loreto, Italy) for 20 s (10 s for occlusal side and 10 s for gingival side) [20]. 

The brackets of Groups 1 (No MR I—No wire), 2 (1.5 T MRI—No wire) and 7 (1.5 T MRI—No wire) served as control groups, and no wire was secured. In the other groups (3 to 6 and 8 to 11), different wires were tested (corresponding to the various materials and dimensions mostly used in orthodontics)—stainless steel (stainless steel wire, Ormco, Glendora, CA, USA) and nickel titanium (nickel titanium wire, Ormco, Glendora, CA, USA) alloys in two different shapes: round (0.014 inch) and rectangular (0.019 × 0.025 inches). Wires were secured in the bracket slots with elastomeric ligatures (elastomeric ligatures, Leone, Sesto Fiorentino, Italy). Specimens were then stored in a physiological solution.

### 2.2. Temperature Test and MRI

Specimens were left at room temperature for 12 h. For each tooth, the temperature of the bracket and the wire was measured in Celsius with a contact thermometer (PeakTech^®^ Digital Thermometer 5135/5140 Prilf und Messtechnik GmbH, Ahrensburg, Germany). The bracket temperature (Groups 2 to 11) was measured by contacting the thermometer probe with the upper right brace wing. The wire temperature (Group 3 to 6 and 8 to 11) was measured by contacting the thermometer probe with the vestibular wire surface, 2 mm mesial to the bracket slot. Temperature measurements were performed immediately before (T0) and after (T1) the MRI exam.

Group 1 served as the control and was not submitted to any MRI exam.

Groups 2 to 6 underwent MRI at 1.5 T power (Magnetom Symphony Maestro Class 1.5 T, Siemens, Munich, Germany), with the sequences described in Table 2. Different sequences were generated by measuring the spin–lattice relaxation using short repetition and echo times (T1) and measuring the spin–lattice relaxation using long repetition and echo times (T2): T2 weighted turbo spin echo in axial projection (T2W-TSE AXIAL), T2 weighted turbo spin echo in coronal projection (T2W-TSE CORONAL), T2 fluid attenuated inversion recovery in axial projection (T2-FLAIR AXIAL), and T1 volumetric interpolated breath-hold examination in three dimensions fat saturated (T1 VIBE 3D FS). Table 2 reports the main characteristics of the four sequences according to the field of view (FOV), voxel size, slice thickness, slices, time of echo, repetition time, scan time and specific absorption rate (SAR) of the whole body.

Groups 7 to 11 underwent MRI at 3 T power (Magnetom Verso A Tim System, Siemens, Munich, Germany), with the sequences described in Table 3. The sequences selected were: T2 weighted turbo spin echo in axial projection (T2W-TSE AXIAL), T2 weighted turbo spin echo in coronal projection (T2W-TSE CORONAL), T2 fluid attenuated inversion recovery in axial projection (T2-FLAIR AXIAL), T1 volumetric interpolated breath-hold examination in three dimensions fat saturated (T1 VIBE 3D FS), T2 weighted two dimensional fast low angle shot for hemosiderin detection in axial projection (T2W-FL2D HEMO AXIAL), T2 weighted turbo inversion recovery magnitude in axial projection (T2W-TIRM AXIAL), 2 dimensional echo planar with 5 mm slice diffusion in axial projection (EP2D DIFF 5 mm AXIAL), 2 dimensional echo planar with 3 mm slice diffusion in axial projection (EP2D DIFF 3 mm AXIAL), and proton density weighted in axial projection (PDw AXIAL). Table 3 reports the main characteristics of the various sequences according to the field of view (FOV), voxel size, slice thickness, slices, time of echo, repetition time, turbo inversion recovery, scan time, and specific absorption rate (SAR) of the whole body.

The total scanning time was approximately 20 min for each group.

After MRI and temperature measurement, all the specimens were stored in a physiological solution.

### 2.3. Shear Bond Strength Test

**Figure 2:**
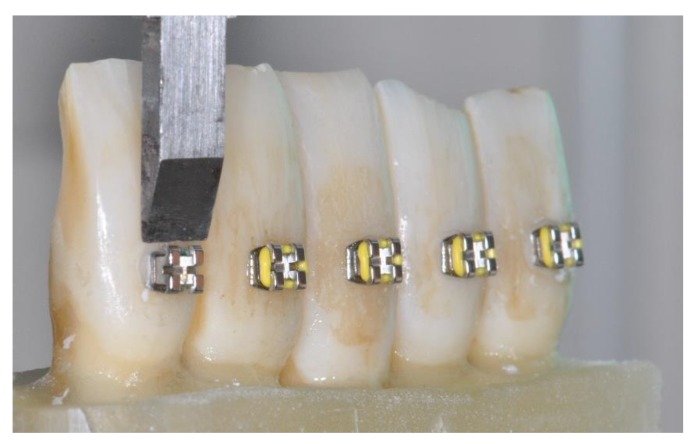
Shear bond strength test. The bracket base was set parallel to the shearing force.

Adhesion strength was measured with a universal testing machine (Model 3343, Instron, Canton, MA, USA). Each resin block containing 10 teeth was sectioned into two blocks of 5 teeth in order to allow insertion into the testing machine. The blocks were included in the mechanical jaw, and the shearing force was parallel to the bracket base [21]. Each bracket was stressed with an occluso gingival force at a crosshead speed of 1 mm/min [22] until adhesive failure (Figure 2).

The maximum load to debond the appliance was recorded in Newtons and subsequently converted into Mega Pascals as a ratio of the force on the surface area [23].

### 2.4. Adhesive Remnant Index Test

**Figure 3:**
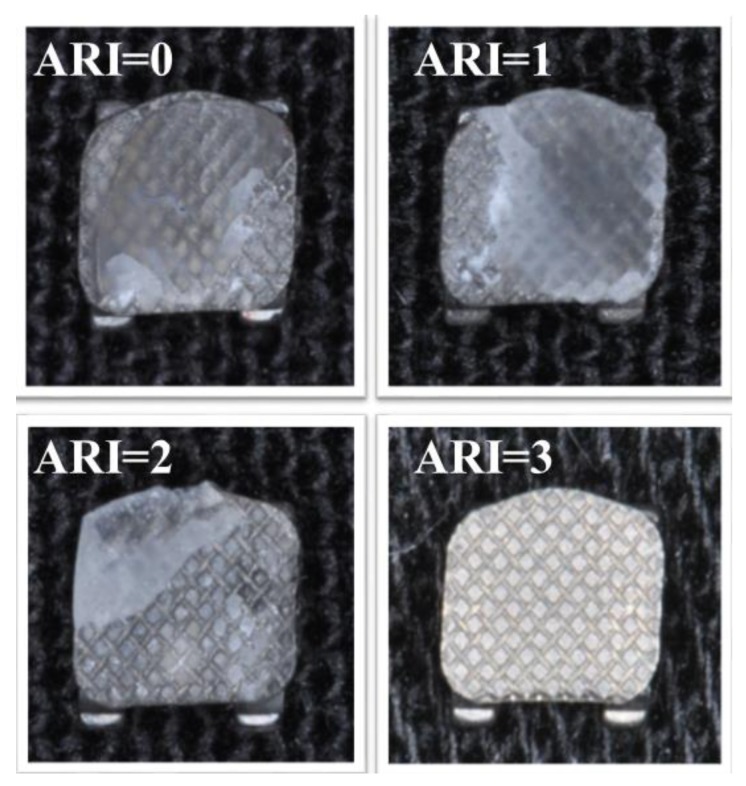
Adhesive remnant index (ARI) score samples. ARI = 0 means all the adhesive is left on the bracket base. ARI = 1 means more than half of the adhesive is left on the bracket base. ARI = 2 means less than half of the adhesive is left on the bracket base. ARI = 3 means no adhesive is left on the bracket base.

After debonding, all the specimens were observed under optical microscopy (Stereomicroscope SR, Zeiss, Oberkochen, Germany). Both the enamel and bracket base were evaluated and scored using a 0–3 scale [24]. As shown in Figure 3, this scale is used to define the interface, assigning to each specimen a score of 0 (no adhesive left on the enamel and all the adhesive left on the bracket base), 1 (less than half of the adhesive left on the enamel and more than half of the adhesive left on the bracket base), 2 (more than half of the adhesive left on the enamel and less than half of the adhesive left on the bracket base), or 3 (all the adhesive left on the enamel and no adhesive left on the bracket base).

### 2.5. Statistical Analysis

Numeric analysis of the data was performed using computer software (R^®^ version 3.1.3, R Development Core Team, R Foundation for Statistical Computing, Wien, Austria). Descriptive statistics, including the mean, standard deviation, minimum, median, and maximum values, were calculated for all groups. 

A linear regression model for bracket temperature, wire temperature, and shear bond strength was performed, adding as covariates the wire material, the wire dimension, and the MRI power.

Additionally, the normality of distribution was calculated with the Kolmogorov and Smirnov test. Inferential statistics were performed with ANOVA and Tukey tests for temperatures and shear bond strength values. A Chi squared test was performed for the ARI Scores.

The significance was predetermined at P < 0.05 for all tests.

## 3. Results

Linear regression models (Table 4) showed that the wire and bracket temperatures were significantly affected by the MRI Power (P < 0.0001), wire material (P < 0.0001), and wire size (P < 0.05). The shear bond strengths were not significantly affected by the wire material, wire dimension, or MRI power (P > 0.05).

### 3.1. Temperature Test

**Figure 4:**
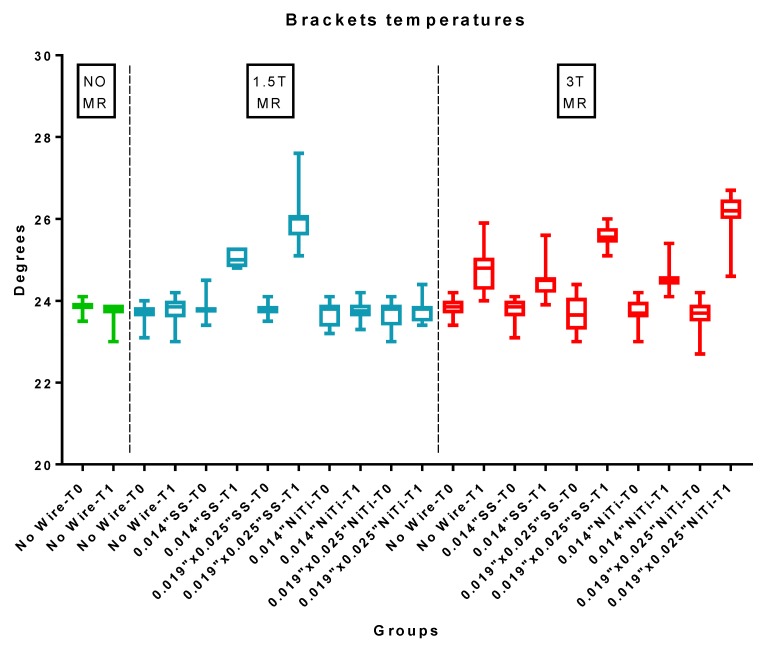
Bracket temperatures (°C) of the various groups before (T0) and after (T1) the 1.5 T and 3 T MRIs. SS: stainless steel wire; NiTi: nickel titanium wire.

Descriptive statistics of the temperatures measured on the brackets are reported in Table 5 and Figure 4.

Additionally, ANOVA showed the presence of significant differences among the various groups (P < 0.05). The Tukey test showed that, when evaluating bracket temperatures after a 1.5 T MRI, no significant increase in the temperature was recorded when the brackets were tested with no wire engaged (Group 2) or when the nickel titanium wire was engaged (Groups 5 and 6) (P > 0.05). On the other hand, a significant temperature increase (P < 0.05) was measured after MRI when testing 0.014″ and 0.019″ × 0.025″ stainless steel wires, with a mean temperature increase of 1.2 °C and 2.2 °C, respectively.

On the other hand, after 3 T MRI exposure, a significant (P < 0.05.) temperature increase of the brackets was reported under all the conditions tested. The highest brackets temperatures were reported when the 0.019″ × 0.025″ stainless steel (Group 9) and 0.019″ × 0.025″ nickel titanium (Group 11) wires were engaged, with a mean temperature rise of 1.94 °C and 2.39 °C, respectively. No significant differences were reported between them (P > 0.05). Significantly lower (P < 0.05) bracket temperatures were recorded when no wire was used (Group 7) and when 0.014″ stainless steel (Group 8) and 0.014″ nickel titanium (Group 10) wires were engaged, with a mean temperature rise of 0.97 °C, 0.69 °C, and 0.77 °C, respectively. No significant differences were reported among them (P > 0.05).

After the MRI exams, no significant differences were reported between the 1.5 T and 3 T powers in terms of their bracket temperatures (P > 0.05), except when the 0.014″ nickel titanium and 0.019″ × 0.025″ nickel titanium wires were engaged, at which point significantly lower temperatures at 1.5 T were reported compared to 3 T (P < 0.05).

**Figure 5:**
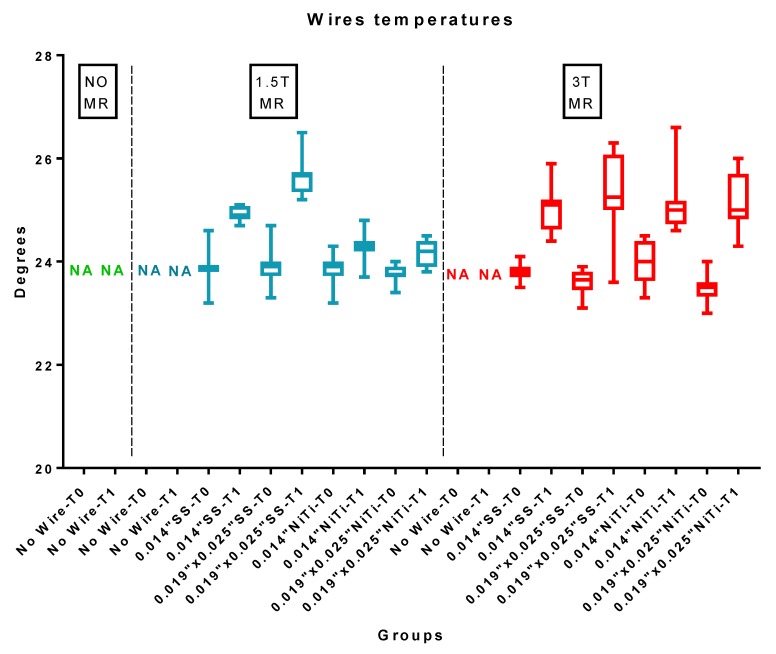
Wire temperatures (°C) of the various groups before (T0) and after (T1) 1.5 T and 3 T MRIs. SS: stainless steel wire; NiTi: nickel titanium wire. NA: not applicable, since no wire was present.

Descriptive statistics for the temperatures of the wires are reported in Table 6 and Figure 5. ANOVA showed the presence of significant differences among the various groups (P < 0.05).

The Tukey test showed that, when evaluating wire temperatures after 1.5 T MRI, a significant temperature increase was reported under all conditions tested (P < 0.05). The highest temperatures (P < 0.05) were recorded for the 0.019″ × 0.025″ stainless steel wires (group 4), which showed a mean temperature increase of 1.69 °C. Significantly lower (P < 0.05) values were reported for the 0.014″ stainless steel wires (group 3), which exhibited a mean temperature increase of 1.01 °C. The lowest values (P > 0.05) were reported for the 0.014″ nickel titanium (group 5) and 0.019″ × 0.025″ nickel titanium (group 6) wires, which showed a mean temperature increase of 0.42 °C and 0.39 °C, respectively, with no significant difference between them (P > 0.05).

Conversely, after 3T MRI exposure, a significant wire temperature increase between T0 and T1 was reported under all the conditions tested (P < 0.05). No significant differences were reported among the various groups at T1 (P > 0.05). The mean temperature increase for the wires was 1.26 °C for the 0.014″ stainless steel (group 8), 1.74 °C for the 0.019″ × 0.025″ stainless steel (group 9), 1.06 °C for the 0.014″ nickel titanium (group 10), and 1.64 °C for the 0.019″ × 0.025″ nickel titanium (group 11) wires.

After the MRI exams, no significant differences were reported between the 1.5 T and 3 T powers for wire temperatures (P > 0.05), except for the 0.014″ nickel titanium wires, which showed significantly lower temperatures at 1.5 T when compared with 3 T (P < 0.05).

When comparing the bracket and wire temperatures, no significant differences were reported at 1.5 T or 3 T exposures (P < 0.05). After the 1.5 T MRI exposure, the mean temperature rise was 0.87 °C for brackets and 0.88 °C for wires. After 3 T MRI exposure, the mean temperature rise was 1.45 °C for brackets and 1.43 °C for wires. When comparing the MRI powers, after 3 T exposure, the temperatures recorded were significantly higher than after the 1.5 T exposure (P < 0.05), with a mean temperature difference of 0.58 °C for brackets and 0.55 °C for wires.

### 3.2. Shear Bond Strength Test

Descriptive statistics of the shear bond strength values are reported in Table 7. ANOVA showed no significant difference in the shear bond strength values among the various groups tested (P > 0.05).

### 3.3. ARI Score Analysis

The frequency distributions of the ARI Scores are reported in Table 8. When no MRI was performed, the specimen showed a significantly higher frequency of ARI = 1 (P < 0.05), whereas after the MRI, all the groups (2 to 11) showed a significant prevalence of ARI = 0, with no significant difference among them regardless of wire size, wire shape, or MRI power (P > 0.05).

## 4. Discussion

The first null hypothesis of the study was rejected. Significant differences in the bracket and wire temperatures were reported. The linear regressions showed significant effects of the MRI power, wire material, and wire size on the temperatures of the orthodontic devices (brackets and wires). Indeed, after MRI, a significant increase in the temperature of the brackets was revealed in all the groups tested, except for the group without a wire and the groups with 0.014″ nickel titanium and 0.019 × 0.025″ nickel titanium wires at 1.5 T, where no significant difference was reported before or after the MRI exam. The mean bracket temperature increase ranged between 0.05 °C and 2.4 °C. Concerning the orthodontic wires tested, all the groups showed a significant increase in temperature after MRI, both at 1.5 T and 3 T. The mean wire temperature increase ranged between 0.42 °C and 1.74 °C.

Previous studies evaluated the effects of 1.5 T MRI on dental materials, showing a mean temperature increase of 1–2 °C for prosthodontic materials [25]. Concerning orthodontic appliances, previous reports evaluated the temperature changes of brackets and stainless steel wires, whereas no studies evaluated the temperature changes of nickel titanium wires with different sizes. Significant changes in temperature were less than 1 °C [26] at 1.5 T, and the temperature rise was higher, in particular, for the metal wire linking the brackets. At 3 T, the temperature increase ranged between 0.1 °C [27] and 2.6 °C [25]. All authors concluded that the increase in the temperature of the stainless-steel devices after MRI was numerically significant but not clinically relevant, as it was limited to a few degrees. These findings are in agreement with the present report. Our research also showed similar results for nickel titanium wires. At present, no study has evaluated the temperature changes of nickel titanium wires of different sizes.

Orthodontic wires are used to provide the required force to move teeth to their correct positions. They can be made of many materials, even if the most commonly used are nickel titanium and stainless steel. The first is capable of exhibiting super-elasticity, which provides light, continuous force for physiological and efficient tooth movement. The second is used for torsional root moment [1,12]. Concerning wire size, the 0.014″ round section is the most frequently used size in the aligning phase, and a 0.019″ × 0.025″ rectangular shape is the most commonly used dimension for sliding mechanics [1,17,19]. Based on these considerations, these two materials and sizes were tested in the present report.

Taking into account the shear bond strength, the second null hypothesis of the present report was accepted. MRI Power, wire material, and wire size had no significant effects on the shear bond strength values. No significant differences in the adhesion values were reported among the various conditions tested. At present, no studies have evaluated the bond strength of orthodontic appliances after MRI. Other dental materials, however, have been investigated [28]. Some authors evaluated the effects of MRI on metal–ceramic restorations, showing that surface roughness increased, and hardness decreased, after 20 min of 1.5 T exposure [29]. Additionally, the authors recommended a Ni-Cr alloy over titanium in the fabrication of metal ceramic frameworks for patients with a recurring need for MRI. Regarding orthodontic devices, a previous study [1] evaluated the tensile strength of 0.016″ stainless steel orthodontic wires after a 1.5 T MRI, showing no significant difference in their mechanical properties. Additionally, some studies evaluated the risk of displacement in magnetic resonance imaging by measuring deflection angles and translational forces. The maximal forces observed were about 0.3 N, and the deflection angles reached 45° in some cases, so the authors considered the risk of detachment and displacement to be non-existent at 1.5 T [26] and 3 T [30] when respecting the usual recommendations. This is in agreement with the present report, which evaluated shear bond strength. The values obtained under all conditions tested in the present report are considered to be clinically acceptable, as they are between 5 and 50 MPa, thereby representing the theoretical limits for an orthodontic biomaterial to sustain masticatory forces without risk of enamel loss [31].

For ARI scores, the third null hypothesis of this study was rejected. The ARI values were significantly lower after MRI, showing a shift from a significant prevalence of ARI = 1, recorded in control group, to ARI = 0, measured in all other groups that underwent MRI. This is probably due to the heating of the metal devices, which seems to modify the amount of adhesive left on the tooth after bracket removal. However, ARI Scores are not directly related to bond strength efficacy [31] but represent a more complex method to assess the failure between the bracket base and enamel. A low ARI Score can be linked to easier polishing procedures, as no adhesive remains on the tooth’s surface [32]. Thus, both the 1.5 T and 3 T MRIs seem to have no negative effects on this variable.

The present report demonstrated that the tested orthodontic materials are safe, based on the variables of bracket and wire temperatures, appliance adhesion, and residual adhesive. However, these devices can have a detrimental effect on the final image quality depending on the anatomical district being studied with the radiologic exam. A classification of dental materials according to their magnetic susceptibility has been proposed [33], and studies have evaluated the effects of orthodontic fixed metal appliances [34] and retainers [35] on image quality. There is fair evidence to suggest that orthodontic devices cause MRI image artefacts both at 1.5 T [8] and 3 T [36]. The removal of metal orthodontic devices prior to MRI is thus recommended, especially if the area of interest is near the appliance [5,37], as in cervical vertebrae, cervical region, paranasal sinuses, and head and neck MRI scans. A brain and temporomandibular joint region MRI should not, however, require the removal of such appliances [36].

A limitation of the present report is that, during an MRI exam, the amount of magnetic field could change significantly depending on the patient’s distance from the radiologic device or the patient’s orientation [38]. However, in our study, orthodontic appliances were positioned in the centre of the MRI device, in the area where the magnetic field was the greatest [1].

Additionally, the results of the present report are reliable only for the materials tested and are not generalizable, as there could be variability in the induced magnetic moments among appliances from different manufacturers. Moreover, clear information on the exact alloy composition of orthodontic appliances is not readily available in most cases [6]. Thus, further in vitro and clinical studies on this topic are recommended in order to give clearer guidelines to clinicians and patients.

## 5. Conclusions

The present report demonstrated the following. A significant increase in temperatures was found for both the brackets and wires in some groups, even if the mean temperature increase was clinically insignificant (from 0.4 to 2.2 °C). The MRI did not condition bracket adhesion in any group, and the ARI Scores were significantly lower after MRI. Therefore, the removal of orthodontic appliances before a routine MRI is not recommended but is suggested only in the case of a void risk or possible interference in image quality.

## Figures and Tables

**Table 1:** Materials tested in the present report.

Material	Commercial Name	Manufacturer	Composition
0.014″ Orthodontic Stainless Steel Wire	Stainless steel wire, 0.014″	Ormco, Glendora, CA, USA	17–20% chromium, 8–12% nickel, and 0.08–0.15% carbon, with iron forming the balance
0.019 × 0.025″ Orthodontic Stainless Steel Wire	Stainless steel wire, 0.019″ × 0.025″	Ormco, Glendora, CA, USA	17–20% chromium, 8–12% nickel, and 0.08–0.15% carbon, with iron forming the balance
0.014″ Orthodontic Nickel Titanium Wire	Nickel Titanium wire, 0.014″	Ormco, Glendora, CA, USA	55% Nickel and 45% Titanium
0.019 × 0.025″ Orthodontic Nickel Titanium Wire	Nickel Titanium wire, 0.019″ × 0.025″	Ormco, Glendora, CA, USA	55% Nickel and 45% Titanium
Orthodontic bracket	Victory MBT	3M Unitek Monrovia, CA, USA	18–20% chromium, 8–12% nickel, and 0.08–0.15% carbon, with iron forming the balance
Orthodontic adhesive	Transbond XT primer	3M Unitek Monrovia, CA, USA	TEGDMA, Bis-GMA, and camphorquinone
Orthodontic paste	Transbond XT resin	3M Unitek Monrovia, CA, USA	Bis-GMA, silane, n-dimethylbenzocaine, and phosphorus hexafluoride, 77% by weight of the inorganic filler (silica)

**Table 2:** Magnetic resonance imaging (MRI) sequences at 1.5 T power (Groups 2 to 6).

-	T2W-TSE AXIAL	T2W-TSE CORONAL	T2-FLAIR AXIAL	T1 VIBE 3D FS
FOV (mm)	210 × 210	200 × 200	235 × 185	200 × 200
Voxel Size (mm)	0.5 × 0.5 × 2.0	0.7 × 0.5 × 2.0	1.2 × 0.7 × 5.0	1.0 × 1.0 × 1.0
Slice Thickness (mm)	2	2	5	1
Slices	24	28	20	128 (slices per slab)
Time of Echo (ms)	84	81	107	2.66
Repetition Time (ms)	3000	3000	9000	6.72
Scan Time (min:s)	6:32	7:20	4:59	6:24
SAR whole body (W/kg)	<0.5	<0.5	<0.5	<0.5

Legend: T2 weighted turbo spin echo in axial projection (T2W-TSE AXIAL), T2 weighted turbo spin echo in coronal projection (T2W-TSE CORONAL), T2 fluid attenuated inversion recovery in axial projection (T2-FLAIR AXIAL), and T1 volumetric interpolated breath-hold examination in three dimensions fat saturated (T1 VIBE 3D FS).

**Table 3:** MRI sequences at 3 T Power (Groups 7 to 11).

-	T2W-TSE AXIAL	T2W-TSE CORONAL	T2W-FLAIR AXIAL	T1 VIBE 3D FS	T2W-FL2D HEMO AXIAL	T2W-TIRM AXIAL	EP2D DIFF 5 mm AXIAL	EP2D DIFF 3 mm AXIAL	PDw AXIAL
FOV (mm)	210	200	235	200	210	210	260	190	210
Voxel Size (mm)	0.5 × 0.5 × 2.0	0.7 × 0.5 × 2.0	1.2 × 0.7 × 5.0	1.0 × 1.0 × 1.0	0.8 × 0.5 × 3.0	0.7 × 0.7 × 2.0	2.2 × 2.2 × 5.0	2.5 × 2.5 × 3.0	0.8 × 0.7 × 3.0
Slice Thickness (mm)	2	2	5	1	3	2	5	3	3
Slices	25	28	20	128	24	24	15	10	25
Time of Echo (ms)	84	81	108	2.14	19.90	57	75	69	9.1
Repetition Time (ms)	3260	3000	9000	6.72	650	5070	7300	4700	3000
Turbo Inversion Recovery (ms)	-	-	-	-	-	220	220	220	-
Scan Time (min:s)	3:24	2:32	3:18	1:39	3:13	2:23	2:55	1:53	2:53
SAR whole body (W/kg)	<0.5	<0.5	<0.5	<0.5	<0.5	<0.5	<0.5	<0.5	<0.5

Legend: T2 weighted two dimensional fast low angle shot for hemosiderin detection in axial projection (T2W-FL2D HEMO AXIAL), T2 weighted turbo inversion recovery magnitude in axial projection (T2W-TIRM AXIAL), 2 dimensional echo planar with 5 mm slice diffusion in axial projection (EP2D DIFF 5 mm AXIAL), 2 dimensional echo planar with 3 mm slice diffusion in axial projection (EP2D DIFF 3 mm AXIAL), and proton density weighted in axial projection (PDw AXIAL).

**Table 4:** Results of the linear regression models of the different variables (shear bond strength, bracket temperature, and wire temperature). The covariates tested were the wire material, wire dimension, and MRI power.

Variable	Coefficients	Estimate	Std. Error	t value	Pr(>|t|)	Confidence Intervals	
						2.5%	97.5%
Bracket temperature	Intercept	0.88	0.07	12.34	<0.0001	0.74	1.02
	WireMaterial	0.54	0.10	5.41	<0.0001	0.35	0.74
	Intercept	0.94	0.07	12.59	<0.0001	0.79	1.08
	WireSize	0.0002	0.00005	4.08	<0.0001	0.0001	0.0003
	Intercept	0.33	0.16	2.07	0.04	0.02	0.64
	Power	0.37	0.07	5.45	<0.0001	0.23	0.50
Wire Temperature	Intercept	0.88	0.07	12.34	<0.0001	0.74	1.02
	WireMaterial	0.55	0.10	5.42	<0.0001	0.35	0.74
	Intercept	0.94	0.07	12.59	<0.0001	0.79	1.08
	WireSize	0.0002	0.0002	4.08	<0.0001	0.0001	0.0003
	Intercept	0.33	0.16	2.07	0.04	0.02	0.64
	Power	0.37	0.07	5.45	<0.0001	0.23	0.50
Shear Bond Strength	Intercept	24.34	0.54	45.23	<0.0001	23.28	25.39
	WireMaterial	0.19	0.76	0.25	0.8	−1.30	1.68
	Intercept	24.68	0.41	60.16	<0.0001	23.87	25.48
	WireSize	−0.00001	0.0003	−0.04	0.97	−0.0007	0.0007
	Intercept	25.66	0.76	33.74	<0.0001	24.17	27.15
	Power	−0.48	0.34	−1.44	0.15	−1.14	0.17

**Table 5:** Descriptive statistics of the temperatures (°C) measured on the orthodontic brackets in the various groups tested (T0: before MRI and T1: after MRI). * denotes Tukey grouping. Means with the same letters are not significantly different.

Group	Wire Size	Wire Material	MRI	Time	Mean	SD	Min	Mdn	Max	ΔT (T1-T0)	Significance *
1	No Wire	No Wire	No MRI	T0	23.84	0.13	23.50	23.90	24.10	-	A
-	No Wire	No Wire	No MRI	T1	23.73	0.25	23.00	23.80	23.90	−0.11	A
2	No Wire	No Wire	1.5 T	T0	23.69	0.27	23.10	23.80	24.00	-	A
-	No Wire	No Wire	1.5 T	T1	23.79	0.28	23.00	23.85	24.20	0.10	A
3	0.014″	Stainless Steel	1.5 T	T0	23.83	0.27	23.40	23.80	24.50	-	A
-	0.014″	Stainless Steel	1.5 T	T1	25.02	0.20	24.80	25.00	25.30	1.20	B
4	0.019″ × 0.025″	Stainless Steel	1.5 T	T0	23.79	0.14	23.50	23.80	24.10	-	A
-	0.019″ × 0.025″	Stainless Steel	1.5 T	T1	25.95	0.52	25.10	26.00	27.60	2.16	C
5	0.014″	Nickel Titanium	1.5 T	T0	23.71	0.30	23.20	23.80	24.10	-	A
-	0.014″	Nickel Titanium	1.5 T	T1	23.76	0.25	23.30	23.75	24.20	0.05	A
6	0.019″ × 0.025″	Nickel Titanium	1.5 T	T0	23.67	0.32	23.00	23.80	24.10	-	A
-	0.019″ × 0.025″	Nickel Titanium	1.5 T	T1	23.76	0.29	23.40	23.80	24.40	0.09	A
7	No Wire	No Wire	3 T	T0	23.84	0.20	23.40	23.85	24.20	-	A
-	No Wire	No Wire	3 T	T1	24.81	0.56	24.00	24.80	25.90	0.97	B
8	0.014″	Stainless Steel	3 T	T0	23.81	0.26	23.10	23.85	24.10	-	A
-	0.014″	Stainless Steel	3 T	T1	24.50	0.42	23.90	24.50	25.60	0.69	B
9	0.019″ × 0.025″	Stainless Steel	3 T	T0	23.66	0.43	23.00	23.65	24.40	-	A
-	0.019″ × 0.025″	Stainless Steel	3 T	T1	25.60	0.24	25.10	25.55	26.00	1.94	C
10	0.014″	Nickel Titanium	3 T	T0	23.73	0.31	23.00	23.70	24.20	-	A
-	0.014″	Nickel Titanium	3 T	T1	24.50	0.30	24.10	24.50	25.40	0.78	B
11	0.019″ × 0.025″	Nickel Titanium	3 T	T0	23.66	0.39	22.70	23.70	24.20	-	A
	0.019″ × 0.025″	Nickel Titanium	3 T	T1	26.05	0.61	24.60	26.20	26.70	2.40	C

**Table 6:** Descriptive statistics of the temperatures (°C) measured on the orthodontic wires in the various groups tested (T0: before MRI; T1: after MRI). * denotes Tukey grouping. Means with the same letters are not significantly different. The significance cut off was set at P < 0.05.

Group	Wire Size	Wire Material	MRI	Time	Mean	SD	Min	Mdn	Max	ΔT (T1-T0)	Significance *
1	No Wire	No Wire	No MRI	T0	-	-	-	-	-		-
-	No Wire	No Wire	No MRI	T1	-	-	-	-	-	-	-
2	No Wire	No Wire	1.5 T	T0	-	-	-	-	-		-
-	No Wire	No Wire	1.5 T	T1	-	-	-	-	-	-	-
3	0.014″	Stainless Steel	1.5 T	T0	23.91	0.33	23.20	23.90	24.60	-	A
-	0.014″	Stainless Steel	1.5 T	T1	24.92	0.13	24.70	24.90	25.10	1.02	B
4	0.019″ × 0.025″	Stainless Steel	1.5 T	T0	23.94	0.35	23.30	23.90	24.70	-	A
-	0.019″ × 0.025″	Stainless Steel	1.5 T	T1	25.63	0.34	25.20	25.70	26.50	1.69	C
5	0.014″	Nickel Titanium	1.5 T	T0	23.89	0.25	23.20	23.90	24.30	-	A
-	0.014″	Nickel Titanium	1.5 T	T1	24.31	0.25	23.70	24.30	24.80	0.42	D
6	0.019″ × 0.025″	Nickel Titanium	1.5 T	T0	23.78	0.14	23.40	23.75	24.00	-	A
-	0.019″ × 0.025″	Nickel Titanium	1.5 T	T1	24.17	0.24	23.80	24.20	24.50	0.39	D
7	No Wire	No Wire	3 T	T0	-	-	-	-	-	-	-
-	No Wire	No Wire	3 T	T1	-	-	-	-	-	-	-
8	0.014″	Stainless Steel	3 T	T0	23.80	0.16	23.50	23.80	24.10	-	A
-	0.014″	Stainless Steel	3 T	T1	25.06	0.47	24.40	25.10	25.90	1.26	B,C
9	0.019″ × 0.025″	Stainless Steel	3 T	T0	23.63	0.21	23.10	23.65	23.90	-	A
-	0.019″ × 0.025″	Stainless Steel	3 T	T1	25.37	0.68	23.60	25.25	26.30	1.74	B,C
10	0.014″	Nickel Titanium	3 T	T0	23.98	0.39	23.30	24.00	24.50	-	A
-	0.014″	Nickel Titanium	3 T	T1	25.04	0.46	24.60	25.00	26.60	1.07	B,C
11	0.019″ × 0.025″	Nickel Titanium	3 T	T0	23.48	0.28	23.00	23.50	24.00	-	A
-	0.019″ × 0.025″	Nickel Titanium	3 T	T1	25.12	0.51	24.30	25.00	26.00	1.65	B,C

**Table 7:** Descriptive statistics of shear bond strength values (MPa) tested at various magnetic resonance imaging (MRI) powers. * denotes ANOVA grouping. Means with the same letters are not significantly different. The significance cut off was set at P < 0.05.

Group	MRI	Wire	Wire Material	Mean	SD	Min	Mdn	Max	Significance *
1	No MRI	No wire	No wire	26.45	4.64	18.44	25.26	33.44	A
2	1.5 T	No wire	No wire	25.07	4.36	16.35	25.13	33.29	A
3	1.5 T	0.014’’	Stainless Steel	24.91	3.56	18.02	24.64	33.81	A
4	1.5 T	0.019’’ × 0.025’’	Stainless Steel	24.49	4.30	18.23	24.85	31.99	A
5	1.5 T	0.014’’	Nickel Titanium	23.33	4.78	15.98	23.02	31.07	A
6	1.5 T	0.019’’ × 0.025’’	Nickel Titanium	25.29	5.14	18.20	24.69	35.43	A
7	3 T	No wire	No wire	24.39	5.56	14.76	24.68	33.95	A
8	3 T	0.014’’	Stainless Steel	24.21	4.71	16.20	24.01	34.62	A
9	3 T	0.019’’ × 0.025’’	Stainless Steel	24.51	5.52	12.04	24.52	33.13	A
10	3 T	0.014’’	Nickel Titanium	24.38	5.44	14.18	23.82	34.83	A
11	3 T	0.019’’ × 0.025’’	Nickel Titanium	24.34	5.24	14.94	24.77	31.85	A

**Table 8:** Frequency distribution (%) of ARI scores (0: no adhesive left on enamel surface; 1: less than half of the adhesive left on the enamel; 2: more than half of the adhesive left on the enamel; 3: all the adhesive left on the enamel).

Group	MRI	Wire	Wire Material	ARI = 0	ARI = 1	ARI = 2	ARI = 3
1	No MRI	No wire	No wire	15	75	5	5
2	1.5 T	No wire	No wire	50	45	5	0
3	1.5 T	0.014’’	Stainless Steel	55	30	10	5
4	1.5 T	0.019’’ × 0.025’’	Stainless Steel	55	40	5	0
5	1.5 T	0.014’’	Nickel Titanium	45	30	25	0
6	1.5 T	0.019’’ × 0.025’’	Nickel Titanium	45	40	15	0
7	3 T	No wire	No wire	55	45	0	0
8	3 T	0.014’’	Stainless Steel	55	35	10	0
9	3 T	0.019’’ × 0.025’’	Stainless Steel	55	45	0	0
10	3 T	0.014’’	Nickel Titanium	50	45	5	0
11	3 T	0.019’’ × 0.025’’	Nickel Titanium	60	35	5	0
